# Identification and expression of genes associated with the abscission layer controlling seed shattering in *Lolium perenne*

**DOI:** 10.1093/aobpla/ply076

**Published:** 2018-12-20

**Authors:** Zeyu Fu, Jiancheng Song, Jiqiang Zhao, Paula E Jameson

**Affiliations:** 1 School of Biological Sciences, University of Canterbury, Christchurch 8140, New Zealand; 2 School of Life Sciences, Yantai University, Yantai 264005, China

**Keywords:** Abscission layer, comparative genomics, gene expression, lignification, *LpSH1*, perennial ryegrass, RT–qPCR, seed shattering

## Abstract

Perennial ryegrass (*Lolium perenne*) is one of the most important pasture grasses in the world. However, seed production is negatively impacted by the seed shattering (shedding) nature of this species. Recently, genes involved in the seed shattering process have been isolated and functionally characterized in several crop species. The aim of this study was to identify the genes playing critical roles in the seed shattering process in perennial ryegrass. DNA sequences of genes involved in seed shattering in the Poaceae were used to identify and isolate target genes in perennial ryegrass using a comparative genomics strategy. The candidate seed shattering genes were identified using an ‘in-house’ perennial ryegrass transcriptome database. The relative expression levels of the candidate ryegrass shattering genes were determined using RT–qPCR during different floret and seed developmental stages. Histological analysis of the abscission layer was also conducted. Homologues of seed shattering genes were identified and isolated from perennial ryegrass, and the relative gene expression results suggested that several genes, including *LpqSH1* and *LpSH1*, might have a role in abscission layer formation during seed development. In addition, lignification of the abscission layer may play an important role in the abscission process. A genetic model for seed shattering in perennial ryegrass is suggested and may be useful for directing gene editing towards the production of a reduced-shattering ryegrass.

## Teaser

Perennial ryegrass (*Lolium perenne* L.) is a key pasture grass in many parts of the world. However, as a relatively undomesticated species, seed production is negatively impacted by seed shattering (shedding) during harvest. We used a comparative genomics strategy and isolated eight genes previously linked to seed shattering in several monocot crop species. Quantitative expression profiling indicated that SHATTERING1 (SH1) is likely to be involved in seed shattering in perennial ryegrass. We suggest that LpSH1 could be targeted via mutagenesis or gene editing to reduce seed shattering in perennial ryegrass.

## Introduction

Perennial ryegrass (*Lolium perenne*) is one of the most important forage grasses in the world (Wilkins 1991). Ryegrass is reported to be the most valuable plant species in New Zealand, with a $14.5 billion impact on New Zealand’s GDP in 2012, due predominantly to perennial ryegrass being a highly valued forage for the livestock industry (Nixon 2016). Consequently, the supply of high-quality perennial ryegrass seed is a significant issue for the agronomic sector. However, there are several factors affecting seed yield in this species, including poor pollination and seed set (Charlesworth 1989), impact of biotic and abiotic stresses (Hampton *et al.* 2016) and large harvest losses (Jensen 1976). The primary cause of harvest loss is seed shattering, i.e. the shedding of seed before and during the harvest process (Elgersma *et al.* 1988). Seed shattering may lead to a seed yield loss of up to 75 % in grass species including perennial ryegrass (Simon *et al.* 1997). Consequently, seed shattering can result in considerable economic losses.

Seed shattering is a developmentally programed abscission event, occurring in a specific tissue, the abscission layer. Genes preventing seed shattering were generally dominant in the early progenitors of crops such as rice and sorghum (Konishi *et al.* 2006; Li *et al.* 2006; Lin *et al.* 2012). During domestication, non-shattering homozygous recessive mutant alleles replaced the dominant alleles as a consequence of unconscious selection over millennia (Konishi *et al.* 2006; Lin *et al.* 2012). A number of the genes responsible for the seed shattering trait have been identified and cloned from several monocot species.

In rice, two significant genes implicated in seed shattering, *qSH1* and *SH4*, have been identified. *OsqSH1* (*QTL of seed shattering in chromosome 1*) is reported to be the major shattering-related QTL in rice (*Oryza sativa*) and has the largest effect on the shattering trait, explaining 68.6 % of the total phenotypic variation in the population (Konishi *et al.* 2006), whereas Li *et al.* (2006) showed that *SH4*, a QTL on rice chromosome 4, explained 69 % of the total phenotypic variance relating to the seed shattering trait within an F2 population derived from a cross between *O. sativa* ssp. *indica* and the wild annual species, *O. nivara*. Their results showed that *SH4* plays a key role in the formation of the abscission layer during the early stages of flower development. However, as the expression level of *SH4* increased until the late stage of seed maturation, *SH4* may also take part in the activation of the abscission process (Li *et al.* 2006). Based on crossing experiments with *qsh1* and *SH4*, Onishi *et al.* (2007) demonstrated that *qSH1* is genetically epistatic to *SH4*.

*Shattering Abortion 1* (*OsSHAT1*) gene, which impacts on the differentiation of the seed abscission layer, plays an essential role for seed shattering in rice (Zhou *et al.* (2012). *OsSHAT1* is an AP2 (APETALA2)-type transcription factor, a putative orthologue of *AtAP2* in arabidopsis. Another AP2 gene previously reported to affect seed shattering is the wheat *Q* gene, which has an impact on a range of characters important for domestication, such as glume shape and glume tenacity (Doebley 2006; Simons *et al.* 2006). Therefore, these two important cereal crops may have analogous mechanisms for the initiation of abscission layers (Zhou *et al.* 2012).

A homologue of *OsqSH1* was identified on chromosome 5 in rice and was named *OsSH5* (Yoon *et al.* 2014). They reported that *OsSH5* was highly expressed in the abscission layer in the pedicels and could enhance the seed shattering trait, but the abscission process still required *OsqSH1*. *OsSH5* was shown to induce another two key genes involved in seed shattering, *OsSHAT1* and *OsSH4*, whose expression was located in the pedicel region of *SH5*-overexpressed lines (Yoon *et al.* 2014). In contrast to *qSH1*, *SH4* and *SHAT1*, which promote abscission layer formation, *SH-H* locates within a 34 kb region of chromosome 7 and acts as a repressor of the seed shattering process in rice (Ji *et al.* 2010). *SH-H* is the only recessive shattering gene so far identified in rice.

In contrast to rice, Lin *et al.* (2012) suggested that seed shattering in wild sorghum (*Sorghum virgatum*) appeared to be controlled by a single gene, *SH1*, which encodes a YABBY transcription factor. They further reported that the genomic regions corresponding to *SH1* were conserved among a number of cereals, including rice (*O. sativa*), maize (*Zea mays*), foxtail millet (*Setaria italica*) and sorghum (*S. bicolor*). Functional analysis also indicated that *SH1* could play similar roles in the abscission process in different monocot species such as sorghum, rice and maize.

Other transcription factors have also been shown to be involved in seed shattering. The *SpWRKY* gene, encoding a WRKY transcription factor in another wild sorghum (*S. propinquum*), may play a role in seed shattering (Tang *et al.* 2013). The rice liguleless gene (*OsLG1*) is reported to play a critical role in seed shattering (Ishii *et al.* 2013). *OsLG1* encodes an SBP (*SQUAMOSA* promoter-binding protein) domain and controls laminar joint and ligule development.

In contrast to cereal crops, most forage plants, such as perennial ryegrass, are much less domesticated, and have been actively bred for fewer than 100 years (Stewart 2006). Therefore, the seed shattering trait is still retained in most of these forage species. As perennial ryegrass and cereal crops belong to the Poaceae (Jones *et al.* 2002), we hypothesized that homologues of the shattering genes identified in cereal crops may exist in perennial ryegrass and play similar roles in regulating the seed shattering process. In the present work, we carried out a comparative genomics approach to isolate these genes in perennial ryegrass, followed by RT–qPCR to reveal the expression profiles of the putative seed shattering genes during floret and seed development. We also conducted morphological and histological analysis of the abscission process in this species. While there were no non-shattering genotypes of perennial ryegrass available in New Zealand with which to compare the expression of the putative shattering genes, the results of this work provide useful information regarding the genetic mechanism of seed shattering in perennial ryegrass and can be used to provide targets for functional analysis and for plant breeders developing reduced shattering lines of ryegrass.

## Materials and Methods

### Isolation of putative seed shattering genes in perennial ryegrass

Candidate target gene sequences of *SH1*, *LG1*, *SH4*, *qSH1*, *SHAT1*, *WRKY* and *Q* from different monocots were obtained from the NCBI database and published articles **[see****Supporting Information—Table S1****]**. To identify the homologues in perennial ryegrass, DNA sequences of each target gene were aligned using the Clustal X software (Version 1.83) to verify their identity, and were then used as query sequences to BLAST search the in-house perennial ryegrass transcriptome database described in Guo *et al.* (2017). The Neighbor-Joining (NJ) phylogenetic trees for identified perennial ryegrass homologues were constructed using Clustal X software with 1000 bootstrap replicates, using an outgroup sequence from Ginkgo *AP2* to root the tree. The NJ phylogenetic tree was visualized with MEGA 7 software.

### Plant materials

Plant material was collected three times, providing three independent biological replicates for the gene expression study. In the first biological replicate, multiple seeds from perennial ryegrass cv. RI009 were sown in 2 L pots with fertilized soil at the University of Canterbury glasshouses (43°31′48″S, 172°37′13″E). For the first 3 months, the pots were placed outside the glasshouses for vernalization of the seeds and then moved to a glasshouse. Temperature in the glasshouse ranged from 15 °C at night to 25 °C during the day. Plant tissue samples were collected from October to December 2014, as the day length increased from 12.5 h to 15.5 h. For spike samples, whole spikes 1–2 cm in length, 4–8 cm and 10–12 cm were collected **[see****Supporting Information—Fig. S1**]. For seed samples, the spikelets were tagged on the day of pollen shedding and then collected at different days after anthesis. For vegetative tissues, flag leaves and nodes were collected at the heading stage. Roots and leaves were collected from 7-day-old seedlings germinated in a petri dish. Each sample was collected from at least five individual plants to minimize the variation between plants. Tissue samples were immediately plunged into liquid nitrogen and stored at -80 °C until use.

For the second biological replicate, plants of cv. Grasslands Nui were grown in a field plot located west of Christchurch (43°34′00.5″S, 172°26′45.9″E) in 2014. Whole spikelets at different days after anthesis were collected from multiple individual plants. No early stage spikes and vegetative tissues were collected for this replicate. The harvested plant material was immediately placed in liquid nitrogen and stored at −80 °C.

For the third biological replicate, plants of cv. Grasslands Nui were grown at Yantai University (37°28′28″N, 121°27′27″E), China. Seeds were sown in a glasshouse in February 2015. One-month-old plants were transplanted to a field plot outside the glasshouse for vernalization. Tissue samples were collected from May to June 2015, as the day length increased from 13.5 to 14.5 h. Tissue types and developmental stages were the same as those for the first biological replicate.

For each of the three biological replicates, the dissection of the abscission layer was carried out slightly differently with details shown in **Supporting Information—Table S2**.

### RNA isolation and cDNA synthesis

Total RNA was extracted from up to 100 mg of frozen samples using the RNeasy Plant Mini Kit (Qiagen, Germany) and immediately stored at −20 °C. DNase I (Qiagen, Germany) was used to avoid genomic DNA contamination. Total RNA from flag leaves was isolated by using Ambion TRIzol (Invitrogen, USA) and then purified by using a Qiagen clean-up kit (Qiagen, Germany). The quality and integrity of the isolated RNA were determined by electrophoresis on 1 % (w/v) agarose gels. The concentration was assessed using a NanoDrop-1000 spectrophotometer (Nanodrop Technologies Inc., USA).

For cDNA synthesis for the first and second biological replicates, 1 μg of total RNA, 50 U Expand Reverse Transcriptase (Roche, Germany), 50 pmoles of oligo (dT) primers and 100 pmoles of random hexamer (pdN6) primers were used in a 20 μL reverse transcription reaction. The final reaction mix was incubated at 42 °C for 1 h. For the third biological replicate, the reverse transcription reaction was carried out using cDNA synthesis kit (Clontech, Japan). The cDNA products were diluted 10-fold with Milli-Q water and stored at −20 °C.

### Reverse-transcription quantitative PCR

The expression of the genes of interest was performed using reverse transcription quantitative PCR (RT–qPCR) with a Rotor-Gene Q real-time PCR instrument (Qiagen, Germany). Specific PCR primers were designed for reference genes and target genes, using Primer Premier 6.20 **[see****Supporting Information—Table S3**]. A reaction volume of 15 μL was used for all qPCRs containing 1 μL of 10-fold diluted cDNA, the relevant primers and home-made SYBR Green master mix (Song *et al.* 2012). PCR products were either directly Sanger-sequenced or cloning-sequenced to confirm their identity. At least three technical replicates for each of the three biological replicates were carried out for each sample set. The relative expression of putative seed shattering genes was corrected using two reference genes, *LpElongation Factor* (*LpEF*) and *LpGAPDH* (*LpGAP*), and calculated using modified 2^−∆∆Ct^ method as described in previous studies (Pfaffl 2001; Song *et al.* 2012).

### Isolation of the full length *LpSH1* gene

The structure of the *LpSH1* gene was determined based on the result of multiple alignments of *LpSH1* and its homologues in related monocot species **[see****Supporting Information—Fig. S2****]**. Primers spanning the exon boundary were designed to isolate the intron sequences **[see****Supporting information—Table S4****]**.

Genomic DNA was extracted from perennial ryegrass cv. Glencar as described in Fu *et al.* (2017). The 25 μL PCR reaction contained 0.2 mM of dNTPs, 1.25 U of TransStart *Taq* DNA Polymerase (TransGen Biotech, China), 1× TransStart *Taq* buffer, 0.2 μM of each primer and 200 ng of genomic DNA . The reaction was incubated at 94 °C for 7 min, followed by 35 cycles of 94 °C for 30s, 56 °C for 30s and 72 °C for 1–2 min, depending on the amplicon length, and finally incubated at 72 °C for 10 min. The PCR products were checked on 1 % (w/v) agarose gels. The PCR products were purified and ligated to the pMD18-T Vector (TaKaRa, Japan) and were then sequenced (Sangon Biotech, China). The full length *LpSH1* gene was hand assembled using the exon regions isolated from the transcriptome database aligning with the exon regions of the intron-containing amplicons.

### Morphological and histological analysis of the abscission layer

Spikelets at different developmental stages were collected from two cultivars, Med line 1 (supplied by PGG Wrightson, New Zealand) and Arrow (supplied by Agriseeds, New Zealand). Two cultivars were grown in pots outside at the University of Canterbury glasshouse complex from March 2017. Samples were collected in December 2017.

Spikelet samples from the two cultivars were collected at 0, 14 and 24 days after anthesis (daa) and fixed in a formalin–acetic acid–alcohol solution (formaldehyde: glacial acetic acid: absolute ethanol: H_2_O = 3:5:30:62). After dehydration through an ethanol/tertiary butyl alcohol series and complete infiltration with paraffin, samples were then placed in an embedding ring. Tissue samples were longitudinally sectioned to 10 μm thickness with a microtome (Model RM2165, Leica) and then stained with safranin-fast Green. The samples were protected with a cover slide and then observed under a microscope (Eclipse 80i, Nikon). A stereomicroscope was used to examine the pedicel junctions after detachment of seeds.

## Results

### Isolation of putative seed shattering genes from perennial ryegrass

To isolate putative seed shattering genes from perennial ryegrass, we used as candidate target gene sequences *SH1*, *LG1*, *SH4*, *qSH1*, *SHAT1*, *WRKY* and *Q* from different monocots as query sequences to BLAST search the in-house perennial ryegrass transcriptome database. In total, nine putative sequences were identified, including two for *qSH1* and *SHAT1* and one each for *SH1*, *LG1*, *SH4*, *WRKY* and *Q*.

**Figure 1.**
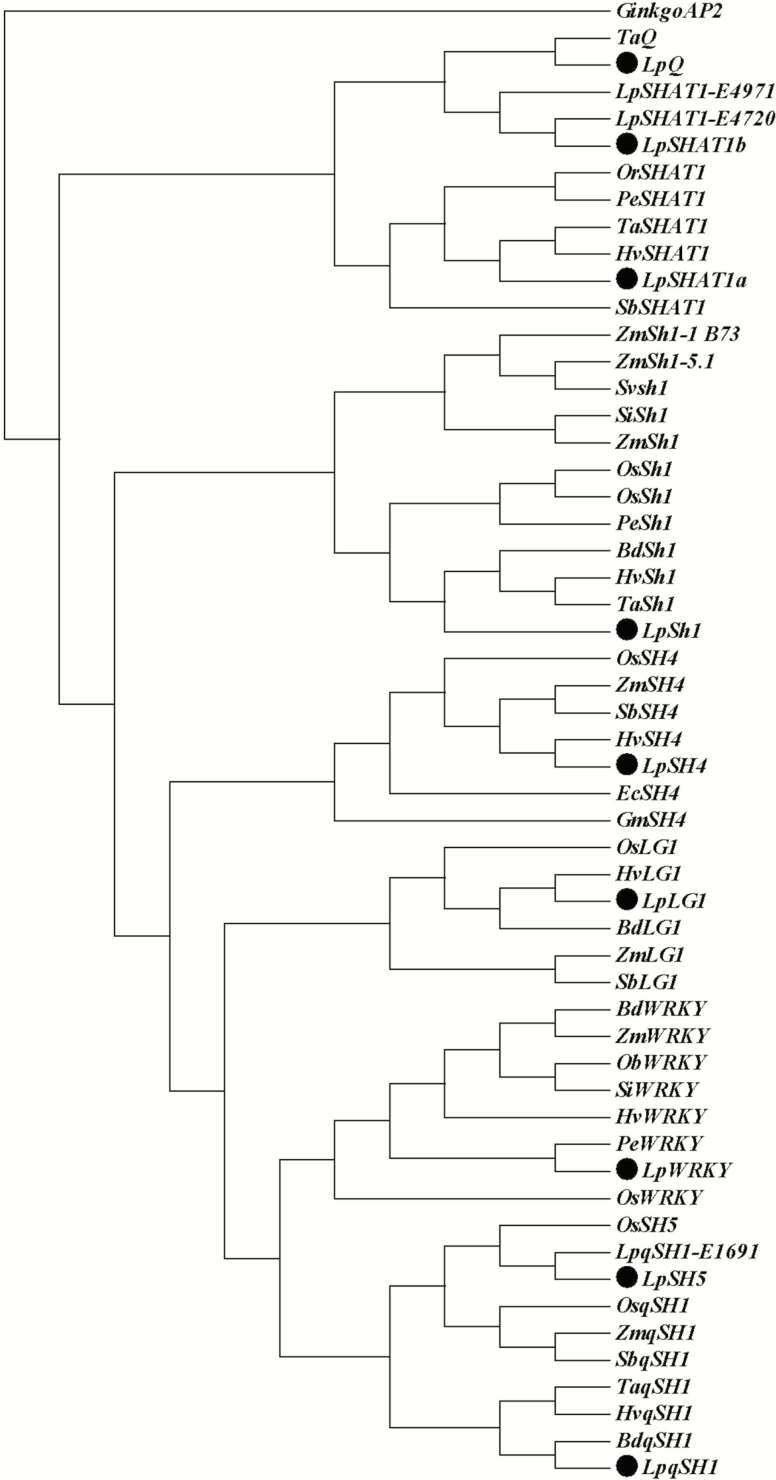
Rooted phylogenetic tree of candidate target seed shattering homologues in monocots. All homologues from perennial ryegrass are marked [·]. The phylogenetic tree was rooted using a Ginkgo *AP2* sequence. Each homologue was renamed for easy reference. Refer also to **Supporting Information—Table S1** for accession numbers. *Ta*, *Triticum aestivum*; *Os*, *Oryza sativa*; *Lp*, *Lolium perenne*; *Or*, *Oryza rufipogon*; *Pe*, *Phyllostachys edulis*; *Hv*, *Hordeum vulgare*; *Sb*, *Sorghum bicolor*; *Zm*, *Zea mays*; *Sv*, *Sorghum virgatum*; *Si*, *Setaria italic*; *Bd*, *Brachypodium distachyon*; *Ec*, *Echinochloa crus-pavonis.*

After the first round identity verification of these sequences through multiple sequence alignment and BLAST searching against the NCBI database, a fragment of each of these sequences was PCR amplified for further sequencing verification. The sequences were named *LpSH1*, *LpLG1*, *LpSH4*, *LpqSH1a*, *LpqSH1b*, *LpSHAT1a*, *LpSHAT1b*, *LpWRKY* and *LpQ*. Accession numbers for these genes are provided in **Supporting Information—Table S1**. A Neighbor-Joining phylogenetic tree was constructed using all of the newly isolated candidate gene sequences and their homologues in related monocot species. The rooted phylogenetic tree is presented in Fig. 1, demonstrating that each putative gene in perennial ryegrass groups together with its corresponding homologues in other monocot species.

*LpqSH1a* is a cDNA fragment of 2432 bp covering the whole coding region sequence of 1824 bp and contains fragments of both the 3′ and 5′ untranslated region (UTR) sequences. *LpqSH1b* is a 1113 bp fragment within the coding region of the gene. LpqSH1a and LpqSH1b belong to BEL1-like homeodomain protein 9, with both containing the key homeobox KN domain (Bürglin 2011). *LpSH1* is a cDNA fragment of 848 bp covering the whole coding region and contains fragments of both the 3′ and 5′ UTR sequences. LpSH1 is a transcription factor and belongs to the YABBY family (Lin *et al.* 2012). *LpQ* is a 1825 bp fragment covering the whole coding region and contains short fragments of both the 3′ and 5′ UTR sequences. *LpQ* translated to a 432 amino acid protein sequence and is an AP2-like transcription factor (Simons *et al.* 2006). *LpSH4* is a 468 bp fragment, with a deduced protein sequence of 134 amino acids from the start point of the coding region and 64 bp of the 5′ UTR sequences. *LpSH4* is a transcription factor, containing a MYB3 DNA binding domain (Li *et al.* 2006). *LpLG1* is a 1003 bp fragment, with a deduced protein sequence of 331 amino acids near the 3′ end of the coding region, and 9 bp of the 3′ UTR sequence. Its protein product is an SBP transcription factor containing a DNA-binding domain (Yamasaki *et al.* 2004). *LpSHAT1a* and *LpSHAT1b* are 1167 and 890 bp fragments, respectively, within the coding region of the genes.

An initially constructed phylogenetic tree showed that *LpqSH1a* and *LpqSH1b* grouped together with *OsqSH1* in rice. The newly isolated *OsSH5* gene is highly homologous to *OsqSH1*, and both genes are reported to play a role in abscission layer formation and differentiation (Yoon *et al.* 2014). Therefore, the realigned phylogenetic tree, after adding the *OsSH5* gene, indicates that *LpqSH1b* (hereafter renamed *LpSH5*) is indeed homologous to *OsSH5*.

In the phylogenetic tree, *LpSHAT1a* clustered to the group of *SHAT1* with other monocot species, suggesting that *LpSHAT1a* is homologous to *SHAT1*. However, *LpSHAT1b* clustered together with two expressed sequence tags from perennial ryegrass, which had a close relationship with *LpQ*, so *LpSHAT1b* was ruled out of the gene list for expression study.

### Relative expression of candidate seed shattering genes in perennial ryegrass

**Figure 2.**
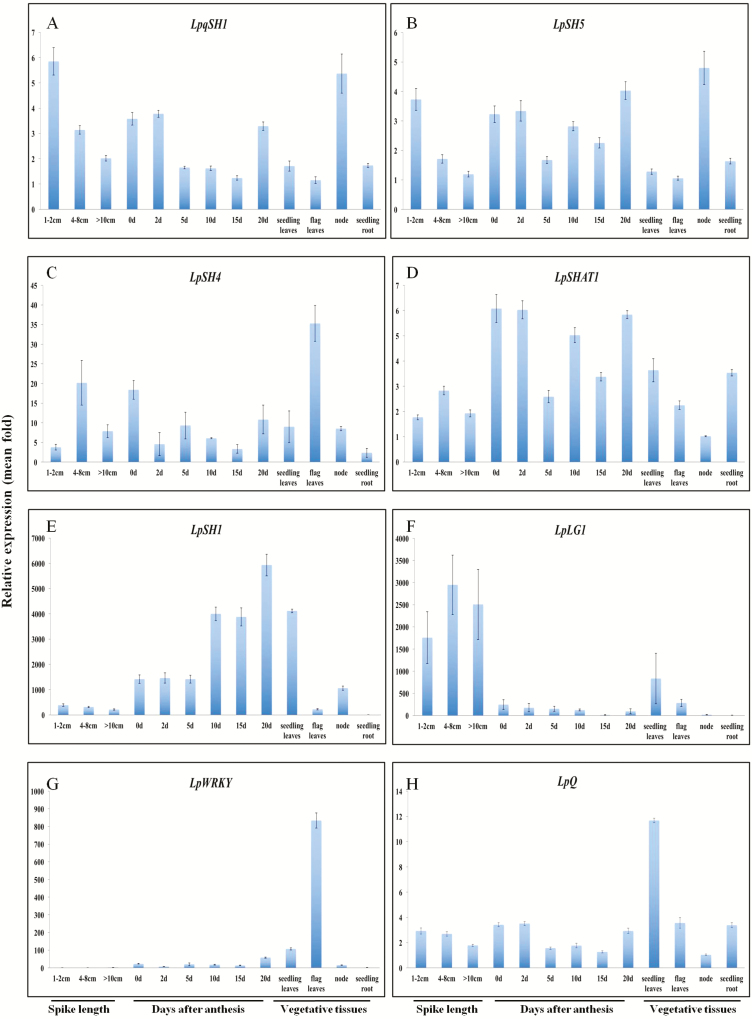
Relative expression of putative seed shattering genes. *LpqSH1*, *LpSH5*, *LpSH4*, *LpSHAT1*, *LpSH1*. *LpLG1*, *LpWRKY* and *LpQ* in perennial ryegrass cv. RI009 (first biological replicate). The plant material was collected from the glasshouse at the University of Canterbury, New Zealand, from October to December in 2014. Values are the mean of three technical replicates (±SD).

To reveal the expression profiles of the newly isolated putative seed shattering regulatory genes, a series of tissue samples including spikes and seeds at different developmental stages, as well as vegetative tissues, were used for RT–qPCR analysis. The expression profiles of eight putative seed shattering regulatory genes are shown in Fig. 2 for the first biological replicate, while those for the second and the third biological replicates are presented in **Supporting Information—Figs S3 and S4**.

Similar expression profiles were detected for *LpqSH1* and *LpSH5* (Fig. 2A and B). At early spike/spikelet developmental stages, these two genes expressed at a high level in spikes of 1–2 cm long, then their expression level declined as spikes developed further. Relatively high expression levels were also detected in early and late seed developmental stages, and in the node (Fig. 2A and B). Strong expression in the node is particularly evident in the third replicate **[see****Supporting Information—Fig. S4****]**.

The expression of *LpSH4* and *LpSHAT1* markedly increased as spikes elongated from 1–2 cm to 4–8 cm (Fig. 2C, D). *LpSH4* showed an increased expression in the late seed developmental stages in the three biological replicates (Fig. 2C, **see****Supporting Information—Figs S3 and S4**).

*LpSH1* was highly expressed post anthesis, in the first biological replicate, from early stages of seed development to the mature seed (Fig. 2E). During spike development, the relative expression level of *LpSH1* was low, but increased markedly around 0 daa, being around 1400-fold higher than the baseline expression. Another increase in expression occurred from 10 to 15 daa, being about 3-fold higher than that at 5 daa (Fig. 2E). A final increase in expression occurred at 20 daa, being 1.5-fold higher than 15 daa, which was 6000-fold higher than the baseline expression (Fig. 2E).

At the early spike/spikelet developmental stages, *LpLG1* had an extremely high expression level compared with its expression during seed development. The expression level of *LpLG1* was also elevated in seedling leaves, but only very low expression was detected in roots and nodes (Fig. 2F). While samples at early spike/spikelet developmental stages were not included in the second biological replicate, a similar expression profile for *LpLG1*was shown for the third biological replicate **[****Supporting Information—Fig. S4****]**.

*LpWRKY* was highly expressed in flag leaves (Fig. 2G, **see****Supporting Information—Fig. S4**) and at mid-seed developmental stages **[see****Supporting Information—Figs S3 and S4****]**.

**Figure 3.**
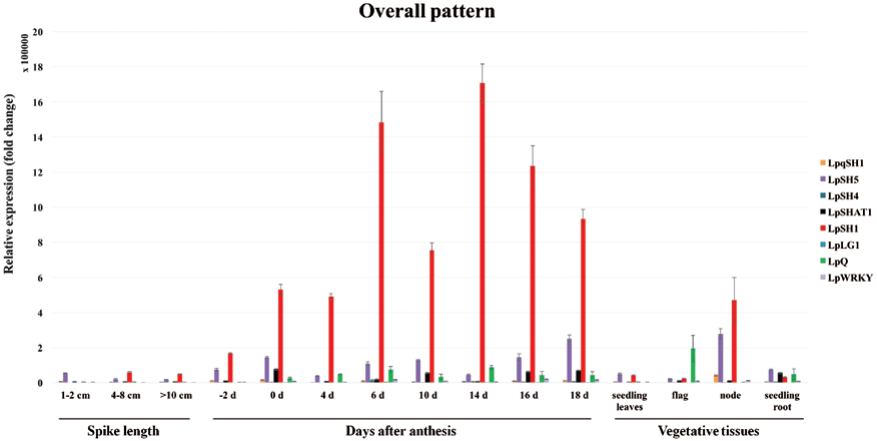
The overall pattern of relative expression levels of candidate seed shattering genes *LpqSH1*, *LpSH5*, *LpSH4*, *LpSHAT1*, *LpSH1 LpLG1*, *LpWRKY* and *LpQ* from perennial ryegrass cv. Nui (third biological replicate). The plant material was collected from field plots at Yantai University campus, China, from May to June in 2015. The relative expression levels are calculated relative to the lowest expressed sample within the same experiment.

From the overall relative expression patterns across the three biological replicates, the expression pattern of *LpSH1* presented the most interesting changes (Fig. 3, **see****Supporting Information—Figs S5 and S6**). The transcript level of *LpSH1* increased during spike/seed development. At the early stages of abscission layer development, *LpSH1* expression was low compared with several of the other genes. However, at later stages, *LpSH1* expression was much greater than that of the other candidate genes (Fig. 3, **see****Supporting Information—Figs S5 and S6**).

### Analysis of the full length *LpSH1* gene

**Figure 4.**
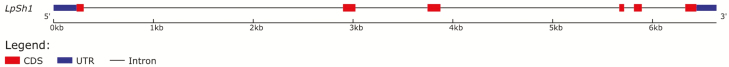
Gene structure of *LpSH1*.

For further analysis of the *LpSH1* gene as a potential target for gene editing, we isolated its Open Reading Frame (ORF) and two untranslated regions from the ryegrass transcriptome database and amplified its intron sequences from genomic DNA. The full-length sequence was verified using TA-cloning and Sanger sequencing **[see****Supporting Information—Table S5****]**. The *LpSH1* ORF contains five introns and six exons. The lengths of the five introns are about 2427, 722, 1792, 98 and 436 bp, respectively. The structure of the *LpSH1* gene is shown in Fig. 4.

**Figure 5.**
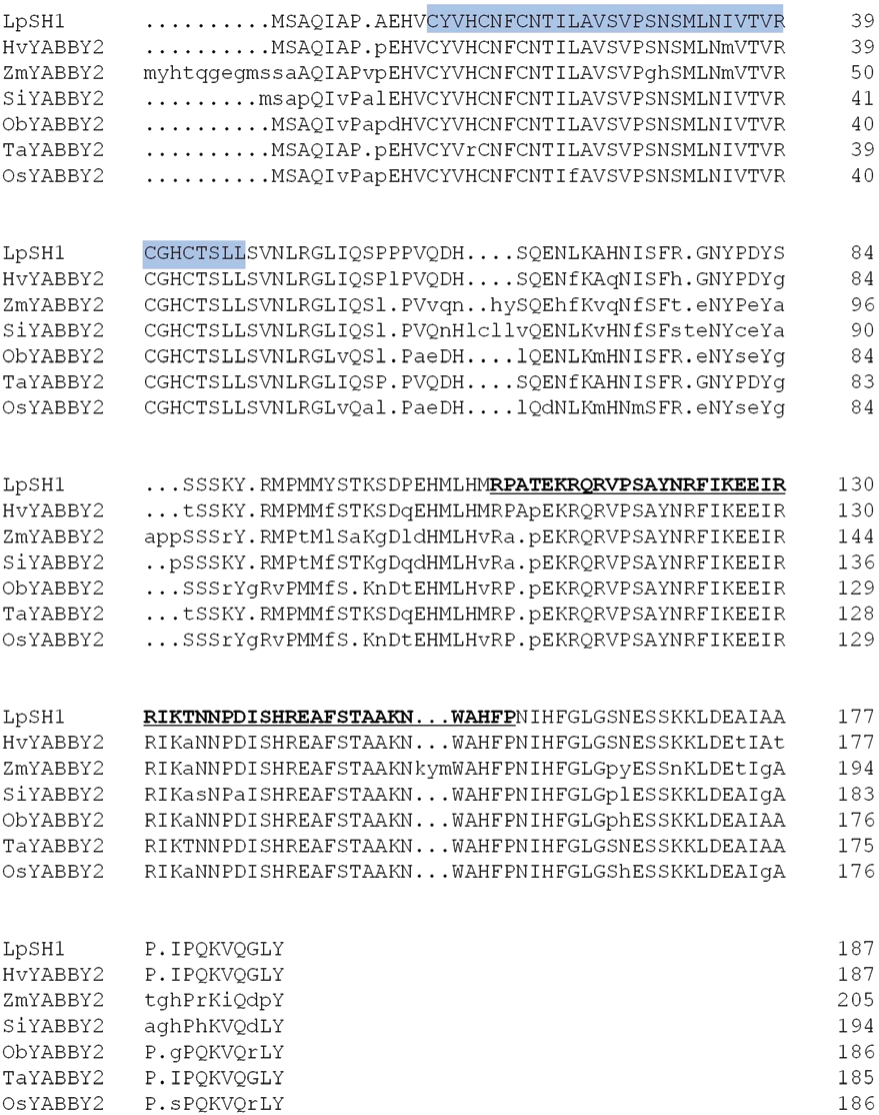
Amino acid sequence alignment of LpSH1 and its homologous proteins. A zinc-finger domain is indicated with the shaded box. The YABBY domain is underlined and in bold for LpSH1. The protein accessions are as follow, HvYABBY2 (*Hordeum vulgare*, BAJ89435.1); SiYABBY2 (*Setaria italica*, XP_004982272.1); ObYABBY2 (*Oryza brachyantha*, XP_006650352.1); TaYABBY2 (*Triticum aestivum*, ABW80974.1); OsYABBY2 (*Oryza sativa*, XP_015628574.1); ZmYABBY2 (*Zea mays*, XP_008666788.2).

The length of the *LpSH1* coding region is 564 bp, encoding a polypeptide of 187 amino acids. The protein sequence contains two conserved domains, including a C2C2 zinc finger domain from amino acid positions 11–47 and a helix-loop-helix YABBY domain between amino acid positions 108 and 156 (Fig. 5) (Lin *et al.* 2012). A phylogenetic tree was constructed using the result of multiple alignments of the amino acid sequence of LpSH1 and its homologues from related monocot species, showing LpSH1 is closely related to other YAB2 subfamily members **[see****Supporting Information—Fig. S7****]**. This suggests that LpSH1 belongs to the YAB2 subfamily. The amino acid sequence of LpSH1 has 93 % identity with the predicted protein HvYABBY (NCBI accession No. BAJ89435.1) and 95 % identity with TaYABBY2 (NCBI accession No. ABW80974). The two conserved domains have high similarity within the YAB2 subfamily of monocot species (Fig. 5).

### Morphological and histological analysis of abscission layer

**Figure 6.**
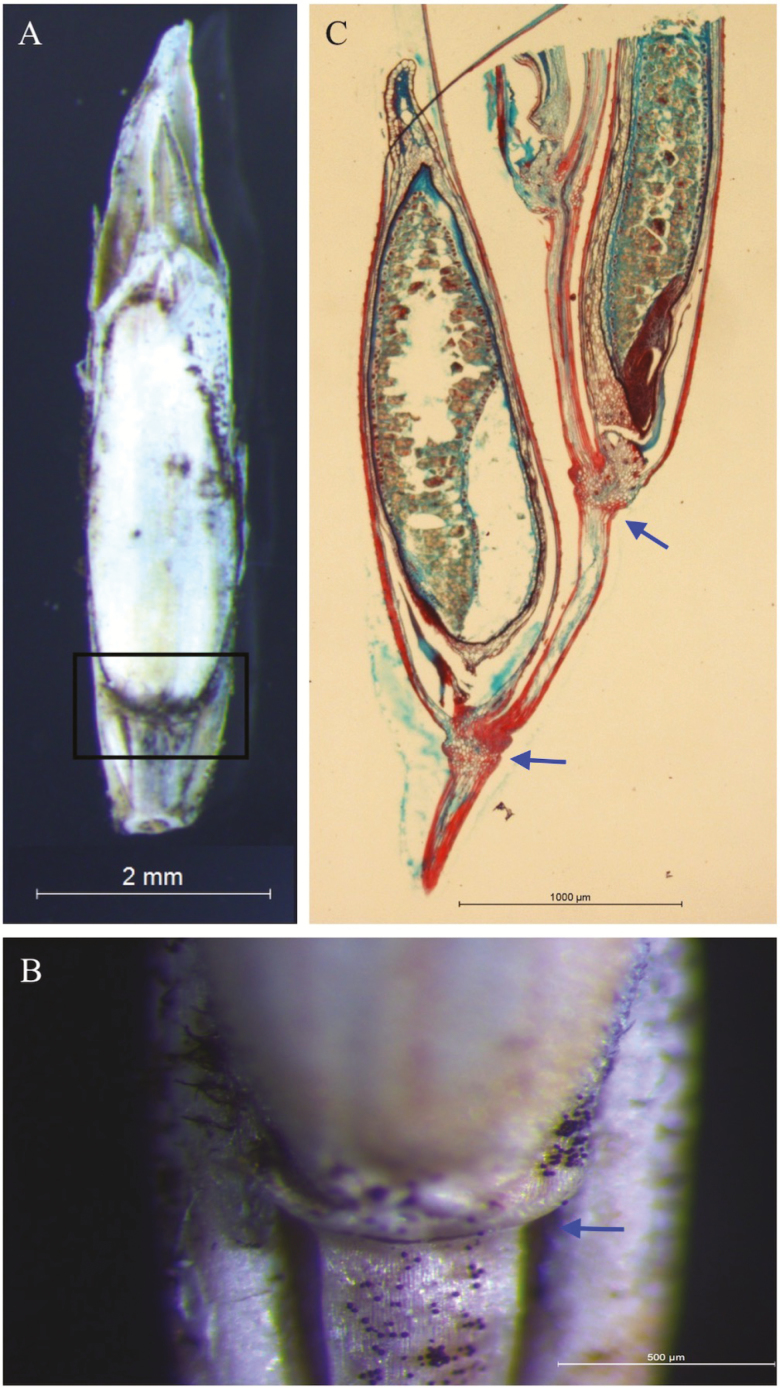
Morphological and histological analysis of the abscission layer at the base of the seed within part of a spikelet. (A) Mature seed on spikelet of perennial ryegrass cv. Med line 1. (B) Enlarged images of the boxed area in (A), showing abscission layer located below the seed, indicated by the blue arrow. (C) Histological longitudinal sections of mature seed on a spikelet collected at 24 days after anthesis. Abscission layers are indicated by arrows.

**Figure 7.**
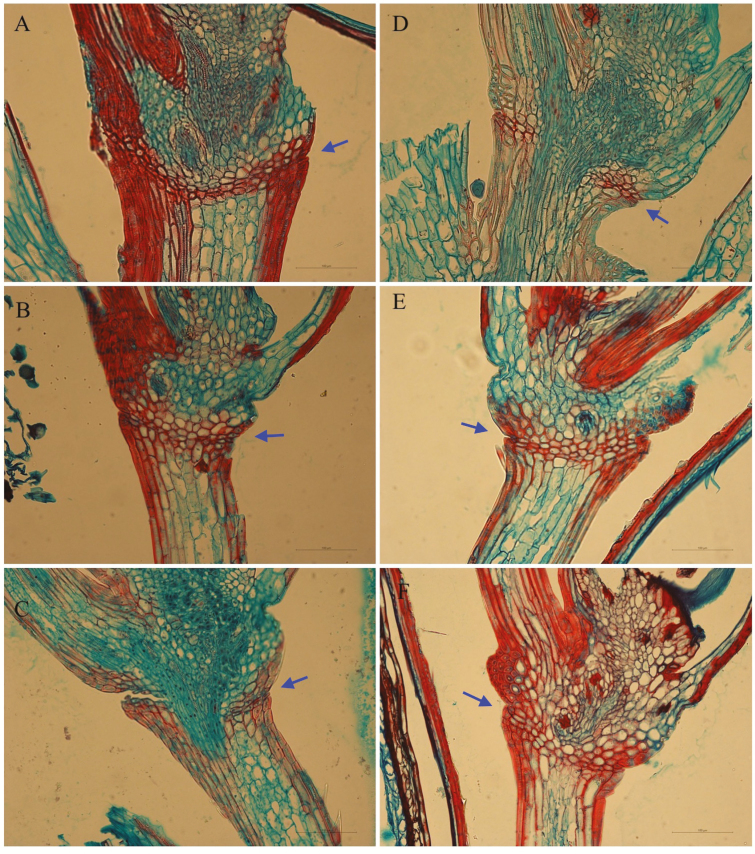
Histological analysis of abscission layer. (A–F) Images of longitudinal sections across the abscission layer at 0 (A and D), 14 (B and E), 24 (C and F) days after anthesis for perennial ryegrass cv. Arrow (A–C) and cv. Med line 1 (D–F), respectively. Sections were stained with safranin-fast green, and the abscission layer is indicated by arrows.

A broken layer can be seen in the spikelet at the base of the floret/seed in cv. Med line 1 (Fig. 6A and B), where seed shattering occurred. This corresponds to the histological analysis of the abscission layer (Fig. 6C). Anatomical investigation of longitudinal sections from different stages of floret/seed indicates that the abscission layer was already present prior to flowering in cv. Med line 1 and cv. Arrow (Fig. 7). The lignin in the abscission layer was stained red by safranin and no change in lignin deposition was observed after flowering in either cultivar. The abscission layer is located in the rachilla below insertion of the floret. The dispersal unit is the floret attached to the distal rachilla internode (Fig. 7). The cells of the abscission layer are smaller than the parenchyma cells in the rachilla and the cells are located around the vascular bundle. The cell walls in the outer area stained more intensely than walls of the cells of the abscission layer in the central area. Dissolution of the abscission layer starts from a notch at the epidermal side.

## Discussion

Comparative phylogenetic analyses have been reported as an efficient strategy to isolate homologous genes and to determine their indicative functions in target species, particularly for species with little genomic information, as is the case for perennial ryegrass (Jones *et al.* 2002; Morrell *et al.* 2012; Song *et al.* 2012). By using a perennial ryegrass transcriptome database and a comparative genomics approach, we isolated eight genes from perennial ryegrass which represent all the previously reported seed shattering genes identified from monocot species including rice, wheat and sorghum. This suggests that perennial ryegrass has a high degree of synteny with these species in terms of genetic control of the seed shattering trait, and that at least some of the candidate genes may potentially share functional similarity to those in rice and other monocot crops. A similar strategy was used by Patterson *et al.* (2016) attempting to identify mutations in *qSH1* in *Digitaria exilis* (fonio), an orphan grain crop in Africa, in which seed shattering is also a serious concern.

Histological analysis in the present study revealed that the location and timing of formation of the abscission layer in perennial ryegrass is the same as that in rice and wild rye (Xie *et al.* 2017; Yoon *et al.* 2017), suggesting that the lignification of the abscission layer in perennial ryegrass plays an important role in the seed shattering trait.

Marked differences in the expression profiles of the putative seed shattering regulatory genes were observed in the current work. For instance, the putative *LpLG1* expressed during panicle development in perennial ryegrass which is similar in timing to the expression of *OsLG1* in rice. *OsLG1* plays a role in panicle architecture, seed shattering and pollination (Ishii *et al.* 2013). This suggests that *LpLG1* may have a similar role in ryegrass panicle structure impacting both seed shattering and pollination. If the panicle structure of ryegrass changed to a spreading panicle, the pollination rate could potentially be increased.

Based on previous studies, *SHAT1*, *SH4*, *qSH1* and *SH5* are involved in abscission layer formation and differentiation in rice (Konishi *et al.* 2006; Li *et al.* 2006; Zhou *et al.* 2012; Yoon *et al.* 2014). More recently, it was demonstrated that a KNOX protein, OSH15, interacted independently with qSH1 and SH5 to induce seed shattering in rice by enhancing abscission layer differentiation (Yoon *et al.* 2017). The expression levels of *qSH1* and *SH5* were not changed in an *osh15* mutant, but the expression of *SH4* was reduced significantly, suggesting that *qSH1* and *SH5* play a role upstream of *SH4* (Yoon *et al.* 2017). Our RT–qPCR results showed that when the length of the spike was only 1–2 cm, *LpqSH1* had a higher transcript level than the other candidate genes, whereas *LpSH4* and *LpSHAT1* expression increased as the spike elongated from 1–2 to 4–8 cm. This indicates that *LpqSH1* possibly also plays a role upstream of *LpSH4* and *LpSHAT1* in perennial ryegrass. In addition, *qSH1* is likely to play a key role in grain/seed abscission in both monocots and eudicots (Zhang *et al.* 2013), and we suggest that this includes *LpqSH1*.

A complex of OSH15-SH5 in rice was indicated to suppress lignin deposition through direct repression of the lignin biosynthesis gene, *CAD2,* which functions in the last step of monolignol biosynthesis (Yoon *et al.* 2017). In addition, Yoon *et al.* (2017) demonstrated that internode development was controlled by OSH15, and our results show that both *LpqSH1* and *LpSH5* expressed highly in the node. As *OSH15* was identified in 2017, the homologue of *OSH15* in ryegrass was not included in this study. However, as both *LpqSH1* and *LpSH5* were identified as expressing at early stage spikelet and node formation, we suggest that a homologue of *OSH15* might also exist in perennial ryegrass and have a similar role in the abscission process. Clearly, lignin deposition plays an important role in abscission layer formation in perennial ryegrass.

The expression of *LpSH4* increased as the seed matured, suggesting that this gene has a role in activation of the abscission process in ryegrass. Both Li *et al.* (2006) and Lin *et al.* (2007) suggest that *OsSH4* takes part in the degradation of the cell wall in the abscission layer. Additionally, *GL4*, an orthologue of *SH4*, was demonstrated to be a key gene with pleiotropic effects controlling seed shattering and grain size in African rice (Wu *et al.* 2017). Introgression lines were generated by using repetitive backcross progeny derived from a cross between wild rice W1411 (*O. barthii*) and cultivated rice IRGC102305 (*O. glaberrima*) with IRGC102305 as the recurrent parent (Wu *et al.* 2017). One introgression line (GIL25) displayed longer and larger seed size, with longer epidermal cells on the outer and inner glumes than that of cultivated rice. Also, GIL25 displayed a complete abscission layer, instead of a deficiency in the abscission layer near the vascular bundle as seen in the cultivated IRGC102305 (Wu *et al.* 2017). Yan *et al.* (2015) identified that a promoter (pSH4) upstream of *SH4* and its multiple *cis*-acting elements, related to its tissue-specific expression. Of them, the abscisic acid response elements involved in the abscisic acid (ABA) hormone signal pathways were identified. They suggested that pSH4 is most likely associated with the formation and activity of the abscission layer, which depends on the presence of ABA (Yan *et al.* 2015). Taken together, LpSH4 probably has an important role in regulating plant cell wall hydrolytic enzymes.

Several candidate genes involved in cell wall degradation have been studied, such as polygalacturonase, cellulase, *OsCel9D* and *XIs* (xylanase inhibitors) in wild rye (*E. sibiricus*) (Zhao *et al.* 2017) and *OsCel9D* and *OsXTH8* in rice (Nunes *et al.* 2014). Of these genes, *OsCel9D* and *OsXTH8* were shown to play an important role in the process of growth and development, such as cell elongation (Rose *et al.* 2002; Zhou *et al.* 2006). These structural genes are probably regulated by SH4 (which is a MYB transcription factor), and this could explain why a SNP mutation in *GL4* in cultivated African rice causes smaller grain size and loss of seed shattering.

In addition to the genes discussed earlier, *SH1*, which was first identified in sorghum, was shown to be a key gene in the abscission process. No abscission layer was observed in the *sh1* mutants, suggesting that *SvSH1* has a role in abscission layer formation (Lin *et al.* 2012). In addition, the orthologues of *SH1* in rice (*OsSH1*) and in maize (*ZmSH1-1* and *ZmSH1-5.1*+*ZmSH1-5.2*) were identified from conserved collinearity of genomic regions, and Lin *et al.* (2012) suggested that *SH1* for seed shattering was under parallel selection during the domestication process in crops. However, an expression analysis of *SH1* at different stages of abscission layer development was not performed in either sorghum or rice (Lin *et al.* 2012). In our work, the expression of *LpSH1* was shown to increase after anthesis, and the relative expression level was markedly greater than that of other candidate genes during the seed development stage, after which the expression level slightly reduced. This suggests that *LpSH1* may have a role in activating the abscission process in perennial ryegrass.

The phylogenetic analysis of amino acid sequences of LpSH1 and other homologues suggests that *SH1* is a YABBY-like gene, belonging to the YAB2 subfamily. This gene family generally regulates complex patterning and growth decisions relating to lateral organ development (Bowman 2000), but also functions in regulating the differentiation of cells in some tissues (Toriba *et al.* 2007). *SH1* in sorghum, rice and maize could play a role in the formation of the abscission layer (Lin *et al.* 2012). Other YABBY genes have also been illustrated to have unique functions, for instance, *fasciated* controls carpel number during flower/fruit development in tomato (Cong *et al.* 2008); *Drooping Leaf* regulates midrib formation in the leaves and carpel specification in the flowers of rice (Ohmori *et al.* 2008) and *OsYAB1* plays a role in meristem development and maintenance of stamens and carpels in rice (Jang *et al.* 2004). YABBY genes contain DNA-binding domains known to maintain the expression of genes related to cell wall biosynthesis and lignin deposition (Tang *et al.* (2013). Additionally, a YABBY transcription factor, *ObSH3*, is required for abscission layer development during African rice domestication (Lv *et al.* 2018). Combining previous studies and our gene expression study, we suggest that *LpSH1* plays a critical role in the seed shattering process from abscission layer formation to abscission activation in ryegrass.

**Figure 8.**
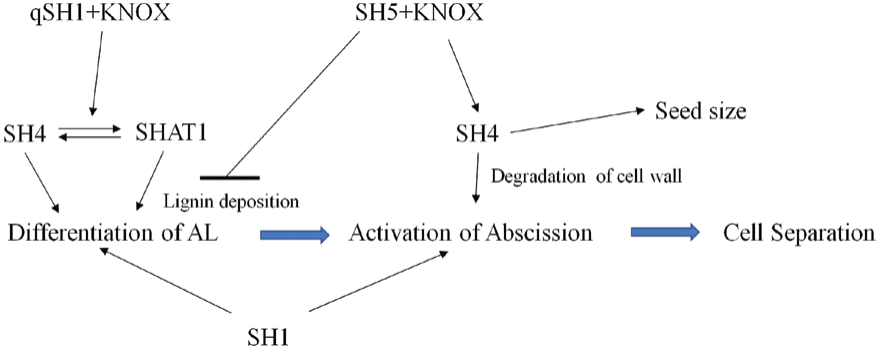
Genetic model for seed shattering in perennial ryegrass.

Based on the earlier discussion, we propose a genetic model for seed shattering in perennial ryegrass (Fig. 8). We suggest that the interaction of LpqSH1 with the KNOX protein OSH15 (Yoon *et al.* 2017) occurs upstream of *LpSH4* and *LpSHAT1*. *LpSH4* and *LpSHAT1* induce the differentiation of the abscission layer in perennial ryegrass. Meanwhile, *LpSH1* could also positively maintain abscission layer formation. It is likely that *LpSH1* continues to have a role during the initiation of abscission, probably by regulating the genes related to lignin deposition (Tang *et al.* 2013). SH5 interacting with a KNOX protein (Yoon *et al.* 2017) could repress lignin biosynthesis and thus inhibit lignin deposition in perennial ryegrass, leading to seed shattering. Additionally, *LpSH5* would maintain *LpSH4* expression leading to degradation of the cell wall to promote abscission through cell wall hydrolytic enzymes. Additionally, *SH4* might have a role in determining grain size and node development (Wu *et al.* 2017).

In conclusion, by using a comparative genomics strategy in combination with a transcriptome data set, we have successfully isolated from perennial ryegrass eight putative seed shattering regulatory genes previously linked to seed shattering in other monocot crop species including rice, sorghum and wheat. Quantitative expression profiles during the shattering time course revealed several genes, particularly *LpSH1*, which could be playing important roles in regulating the seed shattering process in perennial ryegrass. Results of the current work provide useful information relating to the genetic control of seed shattering in ryegrass and provide targets for selection of mutants from TILLING populations or, more directly, for gene editing to reduce seed shattering.

## Sources of Funding

We thank the New Zealand Foundation for Arable Research for the PhD scholarship for Zeyu FU and for project funding, and the National Natural Science Foundation of China (No. 31371616) for J.S. and J.Z. 

## Contributions by the Authors

P.E.J. and J.S. developed and oversaw the project; J.S. provided the perennial ryegrass transcriptome data and analysis; Z.F. grew and collected the samples, conducted all the RT-qPCR and histology, and analysed and interpreted the data as part of his PhD research; J.Z. contributed to the data collection in Yantai. Z.F. wrote the paper with significant input from P.E.J. and J.S.

## Conflict of Interest

None declared.

## Supporting Information

The following supporting information is available in the online version of this article:

**Table S1.** Information regarding seed shattering related homologous genes; GenBank Accession numbers.

**Table S2.** Plant material collected for RNA extraction the three biological replicates.

**Table S3.** Reference and target gene primer sequences selected for the gene expression analysis.

**Table S4.** Selected primer sequences for full length PCR amplification of *LpSH1*.

**Table S5.** The full length sequence of *LpSH1* isolated in this work.

**Figure S1.** The early developmental stages of spikes used for RNA extraction.

**Figure S2.** cDNA sequence of *LpSH1* in perennial ryegrass and comparison of intron length between *LpSH1* and its homologues in several monocot species.

**Figure S3.** Relative expression of putative seed shattering genes for the second biological replicate.

**Figure S4.** Relative expression of putative seed shattering genes for the third biological replicate.

**Figure S5.** The overall pattern of relative expression level of candidate seed shattering genes in the first biological replicate.

**Figure S6.** The overall pattern of relative expression level of candidate seed shattering genes in the second biological replicate.

**Figure S7.** Rooted phylogenetic tree of the YABBY2 sub-family in monocots.

Supplementary Material
