# Supplementary material for: Identification and expression of genes associated with the abscission layer controlling seed shattering in Lolium perenne
Source: AoB Plants. 2018 Dec 20;11(1):ply076. doi: 10.1093/aobpla/ply076 (PMC6343819; doi:10.1093/aobpla/ply076)
Supplement: Supplementary Material [file ply076_suppl_supplementary_material.docx]

**Table S1** Seed shattering-related homologous genes. Accession numbers of *Lolium perenne* sequences identified in this project are in bold.

| Candidate gene | Names used in this work | Species | Accession No./journal |  | Functional description |
| --- | --- | --- | --- | --- | --- |
| SH1 | **LpSH1** | ***Lolium perenne*** | **MK258069** |  | **putative YABBY domain transcription factor family Protein** |
|  | ZmSH1 | *Zea mays* | NM_001148293 |  | putative YABBY domain transcription factor family Protein |
|  | BdSH1 | *Brachypodium distachyon* | XM_003561722 |  | protein YABBY 2-like |
|  | PeSH1 | *Phyllostachys edulis* | FP098112.1 |  | not annotated |
|  | HvSH1 | *Hordeum vulgare* | AK358221.1 |  | not annotated |
|  | TaSH1 | *Triticum aestivum* | AK332816.1 |  | not annotated |
|  | OsSH1 | *Oryza sativa* | EU846982.1 |  | YABBY mRNA |
|  | SvSH1 | *Sorghum virgatum* | Lin et al. (2012) |  |  |
|  | SiSH1 | *Setaria italica* | Lin et al. (2012) |  |  |
|  | OsSH1 | *Oryza sativa* | Lin et al. (2012) |  |  |
|  | ZmSH1-5.1+5.2 | *Zea mays* | Lin et al. (2012) |  |  |
|  | ZmSH1-1 | *Zea mays* | Lin et al. (2012) |  |  |
| LG1 | **LpLG1** | ***Lolium perenne*** | **MK258075** |  | **putative squamosa promoter-binding-like protein** |
|  | OsLG1 | *Oryza sativa* | AB776991 |  | LG1 |
|  | HvLG1 | *Hordeum vulgare* | AM117950 |  | liguleless-like protein |
|  | ZmLG1 | *Zea mays* | NM_001112073 |  | LG1 |
|  | SbLG1 | *Sorghum bicolor* | XM_002447206 |  | protein LIGULELESS 1 |
|  | BdLG1 | *Brachypodium distachyon* | XM_003560343 |  | squamosa promoter-binding-like protein 10 |
| SH4 | **LpSH4** | ***Lolium perenne*** | **MK258074** |  | **putative trihelix transcription factor ASR3-like** |
|  | OsSH4 | *Oryza sativa* | JN697614 |  | shattering 4 |
|  | EcSH4 | *Echinochloa crus-pavonis* | AB455329 |  | sh4 homologue |
|  | ZmSH4 | *Zea mays* | BT069839 |  | not annotated |
|  | SbSH4 | *Sorghum bicolor* | XM_002448695 |  | not annotated |
|  | HvSH4 | *Hordeum vulgare* | AK376279 |  | not annotated |
| qSH1 | **LpqSH1** | ***Lolium perenne*** | **MK258070** |  | **putative BEL1-like homeodomain protein** |
|  | OsqSH1 | *Oryza sativa* | AB071330 |  | qSH-1 |
|  | ZmqSH1 | *Zea mays* | EU973659 |  | not annotated |
|  | SbqSH1 | *Sorghum bicolor* | XM_002458717 |  | not annotated |
|  | BdqSH1 | *Brachypodium distachyon* | XM_003564561 |  | BEL1-like homeodomain protein 9 |
|  | HvqSH1 | *Hordeum vulgare* | AK367206 |  | not annotated |
|  | TaqSH1 | *Triticum aestivum* | AB546647 |  | BEL1-type homeodomain protein |
|  | LpqSH1-E1691 | *Lolium perenne* | GR521691 |  | not annotated |
| qSH5 | **LpqSH5**  OsqSH5 | ***Lolium perenne***  *Oryza sativa* | **MK258071**  Yoon et al. (2014) |  | **putative BEL1-like homeodomain protein**  BEL1-type homeodomain protein |
| Q |  | ***Lolium perenne*** | **MK258076** |  | **putative floral homeotic protein APETALA 2** |
|  | TaQ | *Triticum aestivum* | AY645945 |  | AP2-like transcription factor; Q protein |
|  |  |  |  |  |  |
| SHAT1 |  |  |  |  |  |
|  | **LpSHAT1** | ***Lolium perenne*** | **MK258073** |  | **putative AP2-like transcription factor TOE3** |
|  | Lp-EST | *Lolium perenne* | GR514971 |  | not annotated |
|  | Lp-EST | *Lolium perenne* | GR514720 |  | not annotated |
|  | OrSHAT1 | *Oryza rufipogon* | FO82280 |  | not annotated |
|  | TaSHAT1 | *Triticum aestivum* | AK331198 |  | no annotated |
|  | SbSHAT1 | *Sorghum bicolor* | XM_002447175 |  | no annotated |
|  | PeSHAT1 | *Phyllostachys edulis* | FP093799 |  | no annotated |
|  | HvSHAT1 | *Hordeum vulgare* | AK371953 |  | no annotated |
| WRKY | **LpWRKY** | ***Lolium perenne*** | **MK258072** |  | **putative WRKY transcription factor** |
|  | BdWRKY | *Brachypodium distachyon* | XM_003559570 |  | probable WRKY transcription factor 71-like |
|  | ZmWRKY | *Zea mays* | NM_001151284 |  | putative WRKY DNA-binding domain superfamily protein |
|  | ObWRKY | *Oryza brachyantha* | XM_006651590 |  | probable WRKY transcription factor 2-like |
|  | Hv WRKY | *Hordeum vulgare* | JQ806389 |  | WRKY transcript factor 48 |
|  | OsWRKY | *Oryza sativa* | BK005063 |  | WRKY transcription factor 60 |
|  | PeWRKY | *Phyllostachys edulis* | FP100870 |  | not annotated |
|  | SiWRKY | *Setaria italica* | XM_004982196 |  | not annotated |

**Table S2**. Plant material collected for RNA extraction in three biological replicates.

|  | **Tissue** | **Developmental stage** | **AL collection** |
| --- | --- | --- | --- |
| **the first biological replicate** | **Spike** | Spike length 1-2 cm | Whole spike |
|  |  | Spike length 4-8 cm | Whole spikelet from middle third of spikes |
|  | **Spikelet** | Spike length >10 cm | AL ^a^ |
|  |  | 0 daa |  |
|  |  | 2 daa |  |
|  |  | 5 daa |  |
|  |  | 10 daa |  |
|  |  | 15 daa |  |
|  |  | 20 daa |  |
|  | **Vegetative tissue** | Seedling leaves | N/A |
|  |  | Flag leaves |  |
|  |  | Node |  |
|  |  | Seedling root |  |
| **the second biological replicate** | **Spikelet** | -1 daa | AL ^b^ |
|  |  | 1 daa |  |
|  |  | 4 daa |  |
|  |  | 7 daa |  |
|  |  | 14 daa |  |
|  |  | 18 daa |  |
|  |  | 21 daa |  |
| **the third biological replicate** | **Spike** | Spike length 1-2 cm | Whole spike |
|  |  | Spike length 4-8 cm | Whole spikelet from middle third of spikes |
|  | **Spikelet** | Spike length >10 cm | AL ^b^ |
|  |  | -2 daa |  |
|  |  | 0 daa |  |
|  |  | 4 daa |  |
|  |  | 6 daa |  |
|  |  | 10 daa |  |
|  |  | 14 daa |  |
|  |  | 16 daa |  |
|  |  | 18 daa |  |
|  | **Vegetative tissue** | Seedling leaves | N/A |
|  |  | Flag leaves |  |
|  |  | Node |  |
|  |  | Seedling root |  |

Each sample contained plant tissue collected from multiple individuals

^a^ The top of each floret was cut off, the pedicel junction and the rachilla of the whole spikelet were collected for RNA extraction. daa: days after anthesis.

^b^ Two basal florets of each spikelet were collected and the top of the florets was cut off to collect part of pedicel and rachilla. daa: days after anthesis.

**Table S3**. Reference and target gene primer sequences selected for the gene expression analysis.

| Name |  | Sequences | Amplicon size (bp) |
| --- | --- | --- | --- |
| *LpSH1* | F | AAGAGGCAGCGGGTTCCTTCAG | 189 |
|  | R | GATGGCCTCGTCCAGCTTCTT |  |
| *LpLG1* | F | TGGTGAAGGAAATGCAGGTGGATC | 210 |
|  | R | CAGGCTGTTCTTGCTGCACTTG |  |
| *LpSH4* | F | ACTACCGCAAGGGCAACTGGAC | 190-220 |
|  | R | GTCGTTGCACTGGTTCTGGCTG |  |
| *LpqSH1b* | F | GCTACAAGCAATACTACCAGCAGCT | 221 |
|  | R | CCCGAAGTTCGCCATCTCTTCCT |  |
| *LpQ* | F | AGGAGGAGTTCGTGCACATCCTC | 216 |
|  | R | CTTCCCTCCCRTTGAAGCGAATG |  |
| *LpqSH1a* | F | GGCAGTACTACCAGCAGGTCCAG | 194 |
|  | R | GATGTCTTTGCTCATGCCTTCTTTGAC |  |
| *LpSHAT1a* | F | CGAGGATTACGAGGACATCAAGCA | 210 |
|  | R | TCCCATCTGCCGCACTTGTG |  |
| *LpGAP* | F | AGGAGGTTGCYGTSTTTGGCTG | 237 |
|  | R | GTCAGASKTGTAYTCCTTCTCRTTG |  |
| *LpEF* | F | CACCCTGGTCAGATCGGCAAC | 238 |
|  | R | CACCAACAGCAACAGTCTGCCT |  |

**Table S4**. Selected primer sequences for full length PCR amplification of *LpSH1*.

| Name | Sequences | Region | Expected size |
| --- | --- | --- | --- |
| *LpSH1F3* | CCACATGCACGCACACTTGCACTAG | 5’ UTR | 200bp |
| *LpSH1R4* | GCGAGAATTGTGTTGCAGAAGTTGCAG |  |  |
| *LpSH1F7* | CCGGCGGAGCATGTGTGCTAC | Intron 1 | 2.5kb |
| *LpSH1R6* | GTGGTGGTGACTGGATTAAACCTCTCAAG |  |  |
| *LpSH1F9* | TGCTGAACATCGTGACCGTTCGTTG | Intron 2 | 800bp |
| *LpSH1R8* | GTCACTTTTGGTTGAGTACATCATTGGCATAC |  |  |
| *LpSH1F12* | CCAAATACCGTATGCCAATGATGTACTCAAC | Intron 3 | 1.5kb |
| *LpSH1R10* | GGTTATACGCTGAAGGAACCCGCTG |  |  |
| *LpSH1F14* | GCAGCGGGTTCCTTCAGCGTATAAC | Introns 4&5 | 1.5kb |
| *LpSH1R15* | AGGAATTTAATTTTGGCATTATTCAAACCAGTTCCT |  |  |
| *LpSH1I1F1* | CGTTGTGTAGAGTGTTGCTATTCTTTATCTGAATC | middle part of intron 1 | 500bp |
| *LpSH1I1R1* | TGTGATCTTCGATGTATAGAGAGAGAGATCGAG |  |  |

**Table S5**. The full length sequence of *LpSH1*isolated in this work. The sequences of 5’ and 3’ end UTR are red, exons regions are highlighted, and intron regions are underlined.

CCACATGCACGCACACTTGCACTAGCAATTTGCTCCTATGTGTCTCTCTACTCCATAATTTCTGTATTACACGTTGTACTGATATAAATATCGGACGGCACTCTGACCCGCTAGCTAGTGGTCCTTGGTCTCTTCTTCTACTCCGCCATCTTCCTCATCCTCTCTCCCCTGCCGGCCTTCCCTTTCTTCTTTTGTTCTCGATATCTTGCAACGTCGGGAGGGGAAAAGAATGTCGGCACAGATCGCGCCGGCGGAGCATGTGTGCTACGTGCACTGCAACTTCTGCAACACAATTCTCGCGGTAATATGCCCCATCTCTCTCTCCTCTCTCTCTCTCTCTCATGATTCATGAGGAGATCTACAGAGAGAGATTAGTGGAGCTAATTCTGTAGGATTTGGCTACTTTCTTGCCGAATTTGTGTTTATCAGAGTTACTGGAAAAACGGCTAAGAGATTTATGAATGATTTTGATCCTCTTTTGTTTCGCTACGCCGTGCTCCTTTTGATTTGGCCGCAGCTGAGTTTTCTTATCCGTTTCTACTTGTTTCAAACGAGACTTCGAGGAAGAAAAAGAAAGTGAATGTCAGAACATGTCGTGGAAAATCGATCGAGCTGCTTCTCATCGAATCCATGAAAACCTAGCTATACCTGCTAATCGATTAGTCTCCTGAGCTAATTCACCTCTAGGTATGCTCATGTCATTTGTGGCCTTTGATTTGTCTGACTAATTTCTGAAATTGCATCCCCGCGCCCCCTTTTCTAATTCTTACTGGCCGGGTTTCTTGATGGAACTTGTTGTTAGATCATCGTTGAAATTGTGAAATTGTGAAGTTCATGCCTAGCGATCATGAGTCCATGACAAACTGTACTGTGATGTGTGCAGACTTCATGTAGTAGTACTTGTTATTTTTATTTTCCAAGAACAGCAGCCCTAACCTATTTCAATTGTGGTGGTTTGATTCTAACTAACTCTATTTGAAGACAATTATGGATTTTTTTCTACTTGAATATGCAACACAGAGCATGCAATAACAGTCATAACTTCGGTGTATGATCTATAAAGTTAGCAAGTTAAGTTATTGATCTAAATAGCAATGAAACGTTGTGTAGAGTGTTGCTATTCTTTATCTGAATCAAGTCATGTTTTTTTTCCAGGAAACCGATAGGCTTAAACTATTACACGCAATAGCTAGAACGTAAACTCTTTTATTTATTTATTTATTACTTGAACAGAGGAACAGTAGCATACTGTGTTAGCGAGTTGTCACCTAGAAATTCTGAGTAATGGATGCTTCCTGTCTTCCGCTCTCTCTCTCTCTATATATATATATATCTCCAGTTTGCAGTATGCGTTAATCTGGTTAAAATTGCTACTGGCAATGTGGAGCTTGTGCCTTGGTCTTTTTCCCTCTTTTTTGGGATTTTTGTTGTCCTTGGCATCGAATTAGTACTGTTTGTACTCTTCCTCTCAGTTTCTGCACCTTTATTGTTTGGTTCGTTCATAAGTACATATATATGCATGACACAAGTACTTGTGTGCTGGAGTAATAATTATAGAAAGAAAAAAAGGGAAAAACTAGCTTGATGTACACTATGAACTTGGAGATTGAAGTAATGCCGTCTTGGTCTCTGCTAACCAAGATAAAAAGGAGGTTCAGCTGATATATCTCCCACTCACCAAATGGAATGTTAAACTGATTATGCACAGTGCATCCTGAATTAATTAAACGAGATCTTGCCTTCTCTGGAACTGCAATGCCGTATTTGATCCTCTCTCTCTCTCTCTCTCGATCTCTCTCTCTCTACATCGAAGATCAAAAGGAGAGATGGATCTAGCCTTGTTGACAAACACACCTGCATCTGCGTGACCCACTAAGATGTACTATGCACAGTCTAGGAAACGGCAATGCCGGAATTGAGTCTTTTCTTTTTTTATTCTCGATCTCTCTCTCTATACATCGAAGATCACAAGAGAAGATGAATCTAGCTTGGTGACAAACACACCTGCATCTGCGTGACCCACTAAGATGTACTATGCACAGTAGTGCATGGTGTGGTGCTTATCGTGAGCTAGTCTAGCTAGCGCGCGTGAAGTGCTTTTCTGCATGAACCATCGCTGTCATCTTCCTCTCTTTCCTACAGTGCTTATAGGTGAGATCTTTAATCCTTTTTGCACTGAAGGGAGCATGGAGCAGACATGACGGTAGCGAGCATGGCAGGCCATGGCTAATATTCTTTTGAGAAGAACCCTCACCATACATAATACTGCACGCATCATTCTCTAAGAAATATTTTTCATATATTTTATCTGAAAATAAGCAATTCGAGATATGGTCTGTGATATGAGTCAAGTCCCTTTCATGGGAGCAAACACAATCATCTGCAGATGCATTTGGCTCACTTGCTTATACTACTCTTTCTTATGGGAGTGAGTGCTCAAAAGAAGGAGGTTGAGGAATCAAACAAAGTATAGCTAGAGATACGACCAGCTGGTCCTTCTTCAGGTTGTGGTTGCAGAGCACCCCTGAAAAGAAAACGGCAAGAGTGCAGCCAATGAGCAGGCATCGGAAAAGGTTGCCCCAGTTCTGATTTGGTCCAGCAGAGCCCTGATAAAAGTCATGGAGAGCAACTGTTTTACAGCAGGCACGAGTTATTTATATAGAGAGAGAGAGAGAGAGAGAGAGAGAGAGAGAGAGAGAGAGAGAGAGAGATGCATATATGTGTGTAGATCTGCACCAGCCTTTTGAAAATGCTTGGTTGGAGTTTAGCTCTTTGGTACTTCACTCAGAGAGACCTGGTTATTTTTCTTCTTCTGTTTCTACCTTTTCCCTTCGTGTTTTCTTCTTCCTTGGATGAACTAAAAAACCTAATTGTGTCTCCTTGTGATCGATCTGCAGGTCAGTGTTCCCAGTAATAGCATGCTGAACATCGTGACCGTTCGTTGTGGGCACTGCACTAGCTTGCTGTCGGTGAACTTGAGAGGTTTAATCCAGTCACCACCACCTGTGCAAGATCATTCCCAGGTTAACATGCATCGTAGTAATAGTTCATGAAAGATCATGTTTGCATGAAATTATCTCACCTAAAGGAAGGTTGAGATGTGTATATATAGCTGTATATATGATATTATATGGTGTTATTCCTAGCTTCCTAAGACAGCTGTAGTGGTTTGCTAATTTGATTTAATCACATTTCAAAACATTGTGTTATATTTATATTATTGGAGATAATACAGTCATGCCTACTGTTTGCATTATTTCTCTCTACTACTACCATAGAGGCTAGAAAGATGGTAAGTTTCACAACTGGAAGGGAGAGGTGCCAAACCATACACCTGGCTAGAGCCAAGCTTACCCTAACTCCTACTCTAGCTTATAGCAGAGAAAAGCCAGCATGCATCGGTCATGTCATGCTTCAACGCTTTATACAGTAAAGACAAACGGCAGCATGCACACTGTTCGCTCACAGGAAAGAGTTTTAATGCTCACATGCAAATACAAAAGTAGTACTACATCTCTAGTGTCGTGTACCTGATCGTATATTGTTATATTTTTTTGTGCGGGTATTTTGTTATTTTCTTTTATTGATTTTGAATAGCAATCTTGCACAGCTAGCTTGCGATGTTTATACATTCCAAATCCTCCCGTGAATATATGTTAGAAGTATTCTTCTGTTCTATGTTGATAATTAAATGATGCTTCAAGTCAATTAATGAATTAACTTTATAGTTTTGCTTGATGGCAGGAGAATCTCAAGGCCCACAATATCAGCTTTCGGGGAAATTACCCTGACTATAGCTCTTCTTCCAAATACCGTATGCCAATGATGTACTCAACCAAAAGTGACCCAGAACATATGCTACACATGCGACCAGTAAGATCATGCATATATATTTTGATATTTCACTTTAGTATGTGAATCGTAACTCTTGTTCTTCCCTCTTGATCAATTTGTATACATAGGTGAATTGCAGTACATGTGTATAGTCTTTTCCTTAGGAAAAATACAAAGTTACACCTTGAACTTTTGTACTCTCAAGCCAACTCATCAAACCGATACAAAAAACCATTTTGGTGGTTTTGAAAAGTGTTTTGCCACAAATATATAATGTTTTTTTTTCGCGAAAACGCAAAAACCTTGCGTTTCGATGCATTGATAGAAAAAGAAGGTTATATGTACAAGTCTAAGGGCGGATGAACACCACACCATACAACTCGACCCAAGAAACAGAAATGCTAATCTAGGGGAGCAAAAAGCGCATGCGCCCTGGGCACCCGTGTCCGCCCATTGGCGCGCCTCAACAAATATATAATGGGTTTCGCCAAACATGTGTCAGAAATTTATCCTGCGAAAAGTATGTATCCAAAAGTGAAGATTGCCACCTCAAGTTAGGCCAAACGGGGGAGATATCGAGCAGAGATGGTACTATTTTGCTGAAATTCTACATGCCAACTATAAATCAGCCACGTATTCGCCCGCAATATAAAAATGGGTTTGTTGTCTAGTTTTTCAAGTTCAAGATGTACTATTTGTAGGTTAGGAGTCCAACCATCAATATAATTTGCACCATTATCTGAGATTTTTTACAAGAAAATCTTTAAAATTGAGCACGGAAGAATAAACTTAATGCAAAGTACGATTGGTGTAGGTATTTACTATAGTAGTTTTTTTTTTGAAAGGTTACTATAGTAGTTTTTATTATGTATACATCTCACATTGCTTTGGTATCTAATGAAAATAAAATGGACTCAAGCATAGAGAAAAATGAATAGAGAAATGAAACTTAAATTTCCTATAAAATATTTTGTAGTACACTTTCATTAACTATTAAGTCCCATTAAGGACATGATCTGAATATCTTGATTCGTGCAAGTCCAAATGGATACAAGAAAAAAAAAGTTACAAATTGATGAAAATATCTTAATAAATTGACTACCGTTATCCACTTTTAAATCACTGTGAGATATGGTCACCGACTTAGCTATTTATGTGTCGGGCATACTCCTTTGAGAAAATACATACCTCTTGCATGAATACGTAAACTTGTCTTTCATGGGTAAAAACATTATACCGCTAGGCATAATCATTATTAAATAACTAACTTTGAGTTTCCATAATATCTAGTGATAATTATTAGGGATTAGTGTGTTTCTAGATATCTATTGTGTTACATCTTGATTAATTTTAGCAAAAGTATGTATGTCCTCCATCTTGGGTGCCGAAGTCTAGAAATGATCCTCAATTCTAAAACCAAGAAAACCAGCTCACTCGATTTGTAAAAACGAATTAATTTGGTTCCTTGTATTTTTTTGGAGTGGTTTTGGTGATCATGTGTCCTTGTTTTGACCAAAACCTAATATAATTAATGGCAAATTTTGACTGTTGACCTTGCCATATGTGCATGCATGTCAACACTTGGCTGATCGAAATCAAAACTAGGCCACTCATGCCAAAACCATAGAAATTTGCATGACGGATTAAGAGTATGCAGTTTTAGAAGCGTAAGGACATACAGTTTTTTTATTTAGGAGCATAGTATATTTCTACACTTACCAATTATAGTTTTTTCAAAGGGAGATACATTTATATCAAGATTCCAACAGTTTGGATAAAAATATATGTGTTTCCCTTAAATTTATGAGGATGAATCTTTGATACTGCAGCAGCTACCGAGAAGAGGCAGCGGGTTCCTTCAGCGTATAACCGATTTATTAAGTAAGTTTCAATCAATTAAATGCAAGCTTGTACTAAACATAATGAAGCGG**TAT**TGTCCTCTACTAACCATATATACCATTCTGACATATAAATTTCAAGGAAGAGATACGGAGGATAAAAACAAACAACCCTGACATAAGCCACAGAGAAGCCTTCAGCACTGCAGCAAAGAACGTTAGTACATGCTACGCATAACACCTATGTTGTACTACCACAGTTTTACATTTGCAGGTCACAACTACTCCAGTTTCGTCCGTCAAAAAAACAAAAAAAAAACTACTCCAGTTTCCACACTTGACCATAACCCATAGTTTTGGTATATAACAAACAACTTTCATATGCATGCATATGCAAAAGAGCCACAAATTAATAGCTAAATCACTCAGATAGAAATTGACGCGTATGTTGCTCTGTCATTTGCCCTGGCCAAAGCTTATATTACTGCAGATAACTCCATTTCTAGGGCATGTTTGGAATACAAACAGAAAATTCCTGTAGATCTCAGATTTTTTTTGTACCTTGTCCCAAAGGGATTCTAGTGTTTCTCTGGAGACAGTTGATACGAAATACTACTGTTTAGTTTACATCTCTCTACTGATTTCTATTTCAGTGGGCGCATTTCCCTAACATACATTTCGGCCTAGGCTCCAATGAGAGCAGCAAGAAGCTGGACGAGGCCATCGCGGCGCCTATCCCCCAGAAAGTTCAAGGTCTCTACTGAGACAAATATTCCACTATGCATGATATTTCATATTGTAAGTAATTTAAGGAAATCTAAGTTATTTCTACATCAGTTTGTGAGTGCGTACATAGATCACTTGCGAGCGGCATGCGTGAAGTGAGAGAAAAAAGCATATGGATGGCTTCTTAATAAATCCCTTTGATCGATCATCCTGTTCTTATCCTGTTTTTTTCTATTAGGAACTGGTTTGAATAATGCCAAAATTAAATTCCAATCTCTAGAGGATCCCCGGGTACCGAGCTCGAATCGTAATCATTCAATGTTCCC


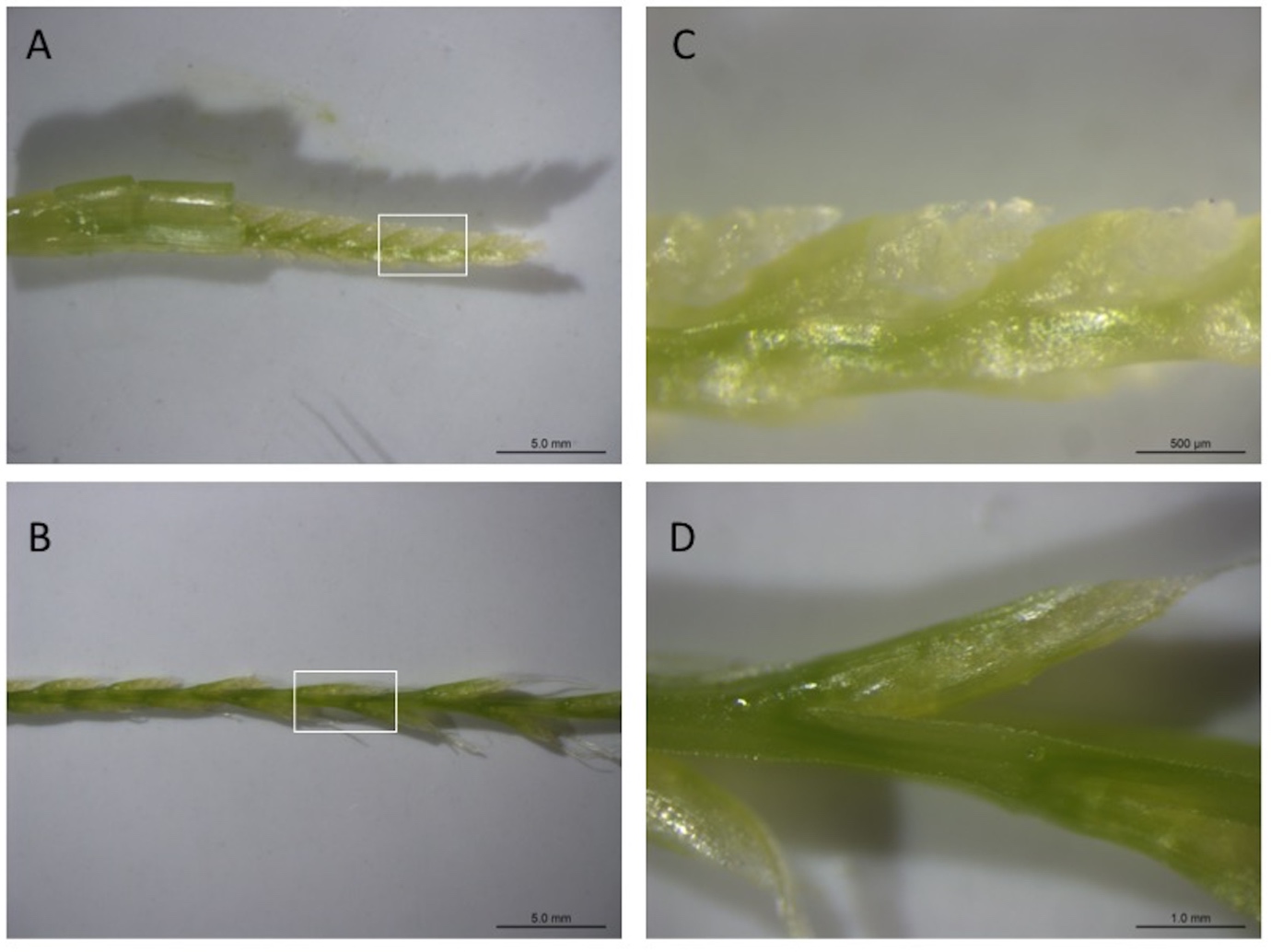


**Figure S1**. The early developmental stages of spikes used for RNA extraction. A: a spike of 1-2 cm long; B: a spike of 4-8 cm long; C, D: enlarged images of the boxed area in the left-hand panels, showing developmental stage of florets on the spikelets.


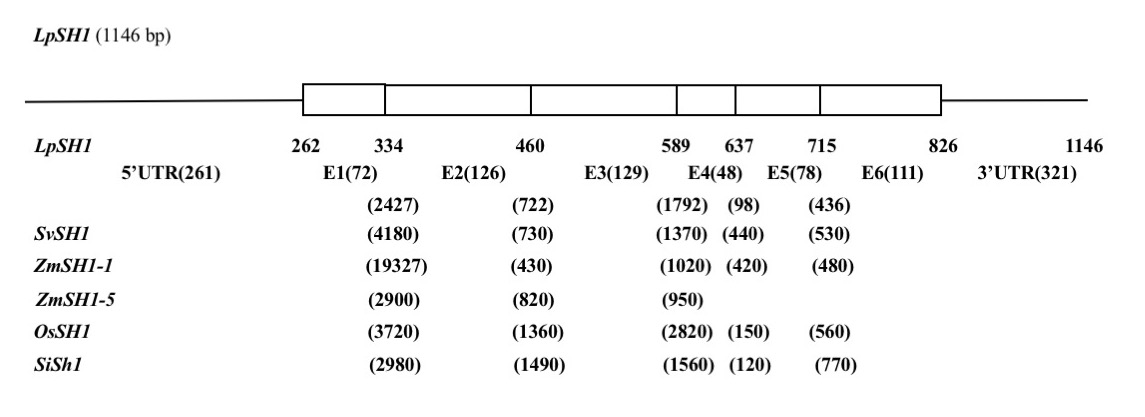


**Figure S2**. cDNA sequence of *LpSH1* in perennial ryegrass and comparison of intron length between *LpSH1* and its homologues in some monocot species.

The cDNA length of *LpSH1* is 1146bp. The untranslated regions (5’UTR and 3’UTR) and six exons (E1-E6) were presented with their lengths and start positions. The intron lengths for *LpSH1* and its homologues in four monocot species were shown in brankets. *Os, Oryza sativa*; *Zm, Zea mays; Sv, Sorghum virgatum; Si, Setaria italic.*


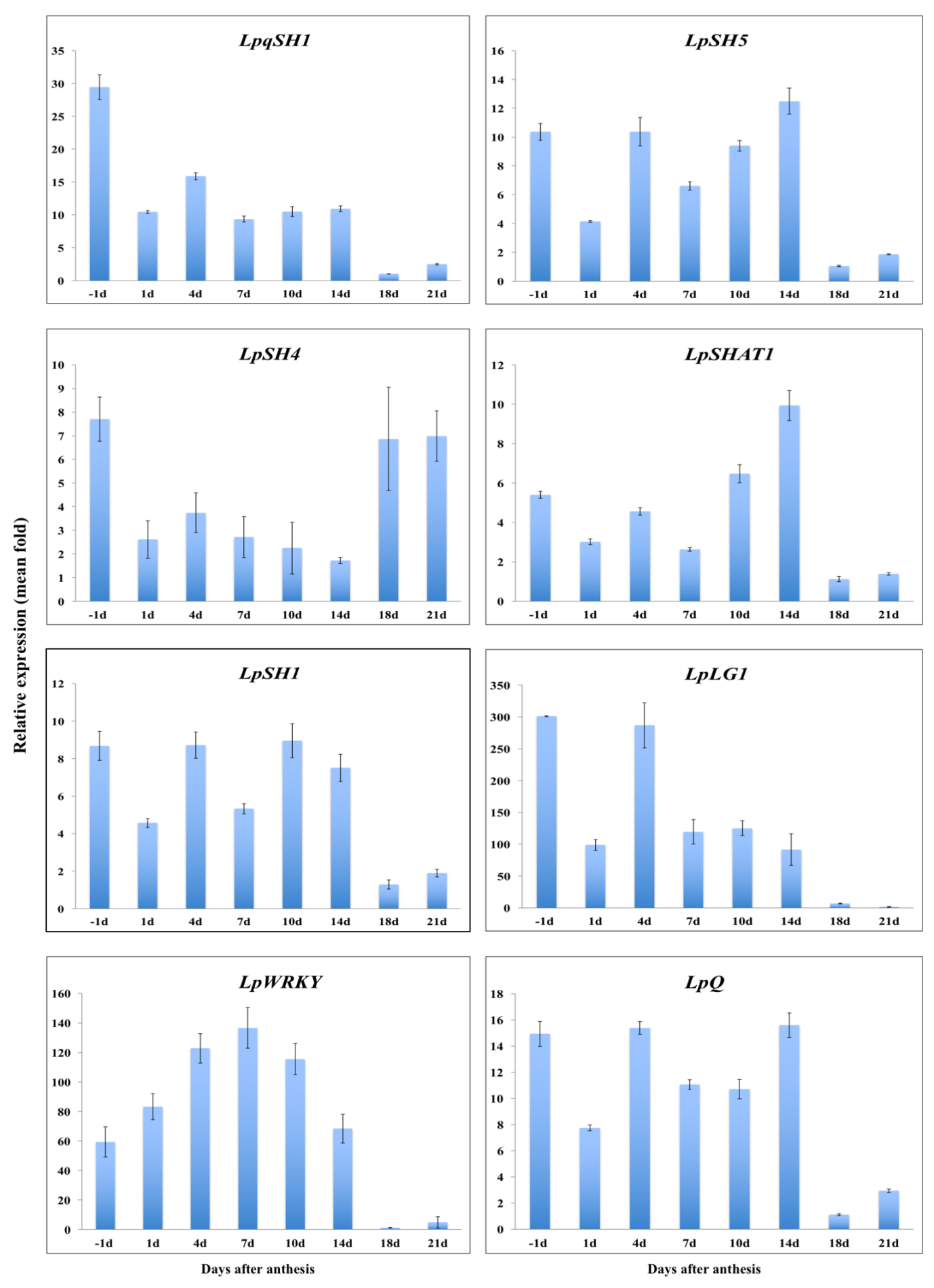


**Figure S3**. Relative expression of putative seed shattering genes for the second biological replicate.

*LpqSH1*, *LpSH5*, *LpSH4*, *LpSHAT1*, *LpSH1 LpLG1*, *LpWRKY* and *LpQ* in perennial ryegrass cv. Nui. The plant materials were collected from a field plot located outside of Christchurch, New Zealand, from October to December in 2014. The values were the mean of three technical replicates ($\pm$SD).


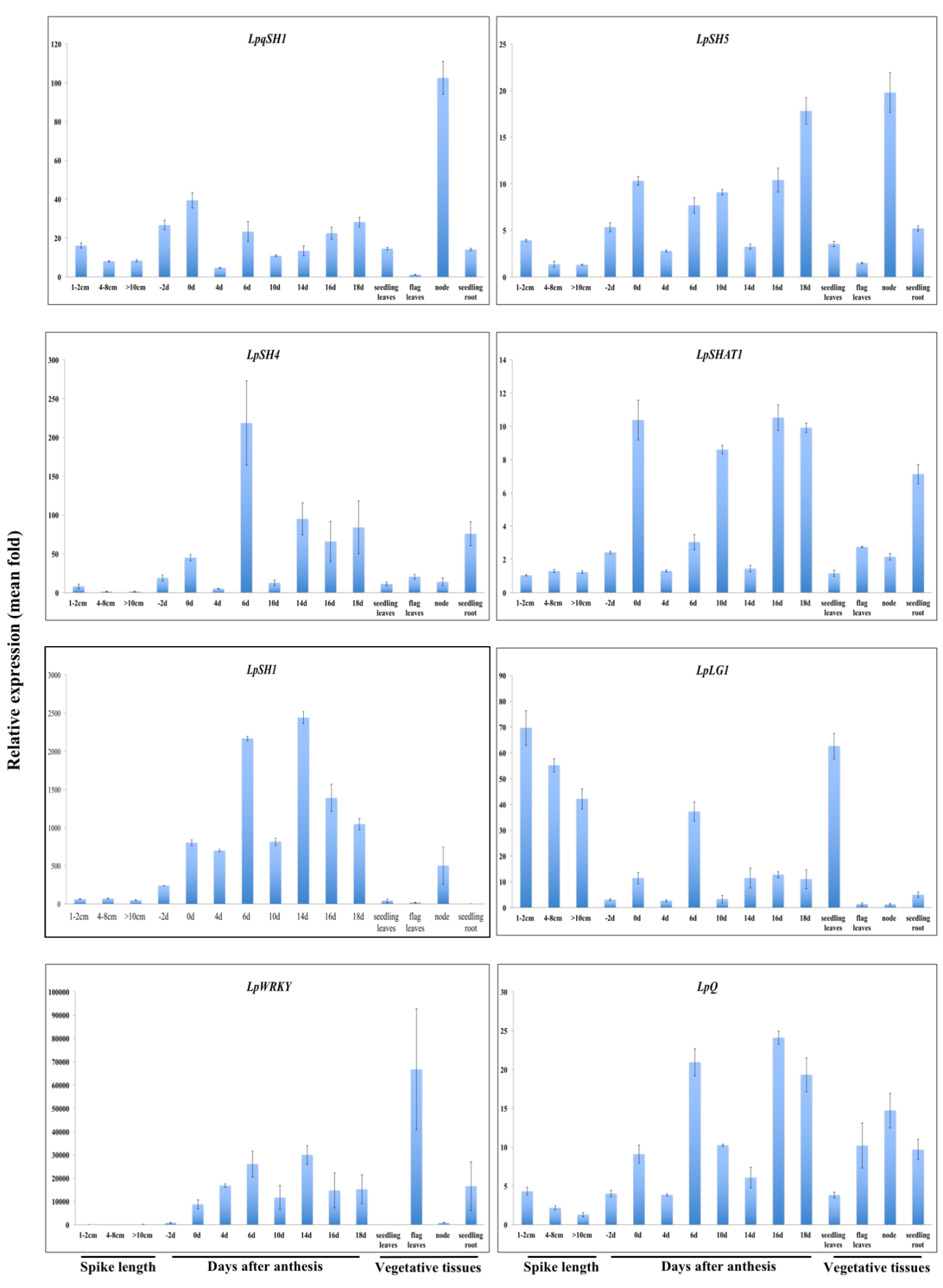


**Figure S4**. Relative expression of putative seed shattering genes for the third biological replicate

*LpqSH1*, *LpSH5*, *LpSH4*, *LpSHAT1*, *LpSH1 LpLG1*, *LpWRKY*, and *LpQ* in perennial ryegrass cv. Nui. The plant materials were collected from field in Yantai University, China, from May to June in 2015. The values were the mean of three technical replicates ($\pm$SD).


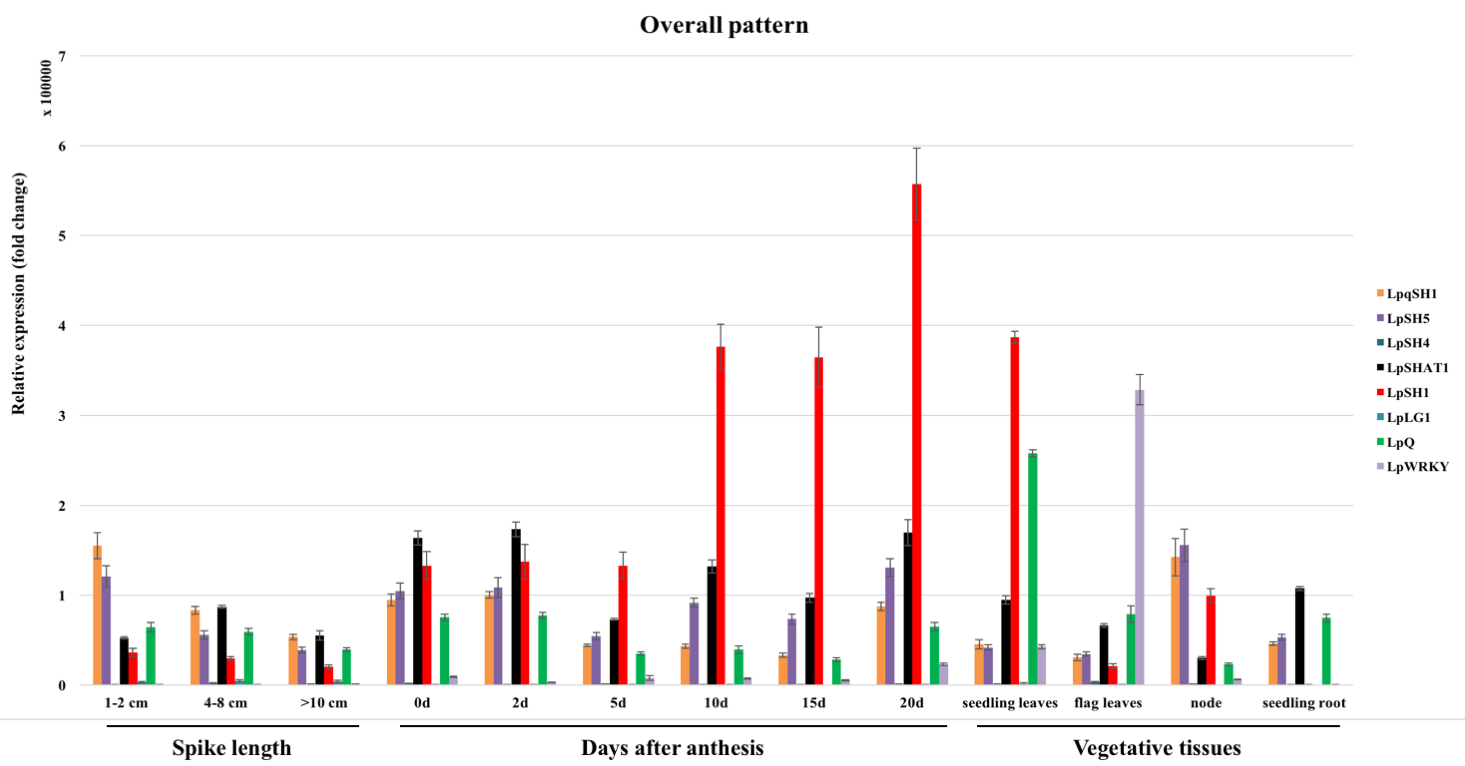


**Figure S5**. The overall pattern of relative expression level of candidate seed shattering genes in the first biological replicate.

The relative expression levels were calculated relative to the lowest expressed sample within the same experiment.


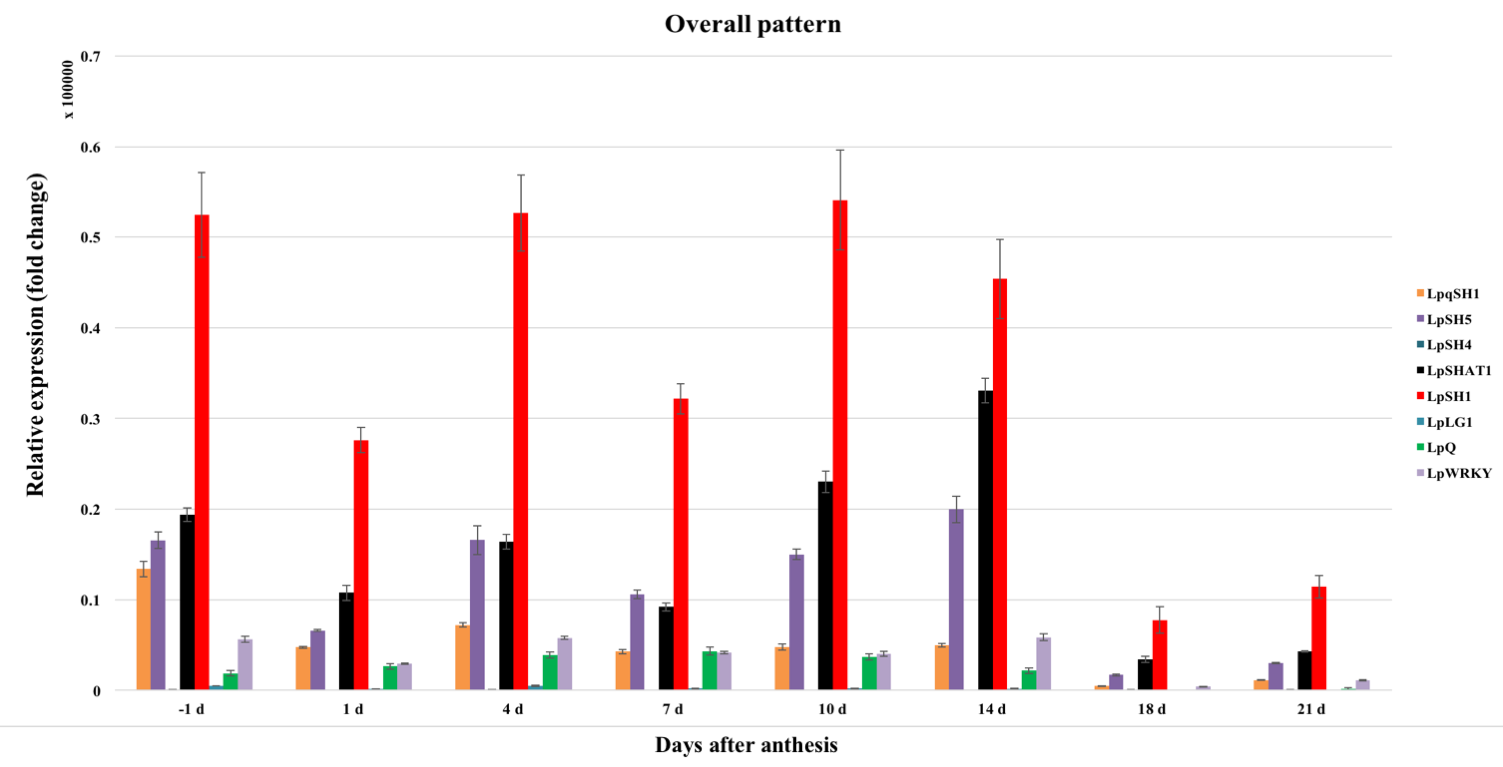


**Figure S6**. The overall pattern of relative expression level of candidate seed shattering genes in second biological replicate.

The relative expression levels were calculated relative to the lowest expressed sample within the same experiment.


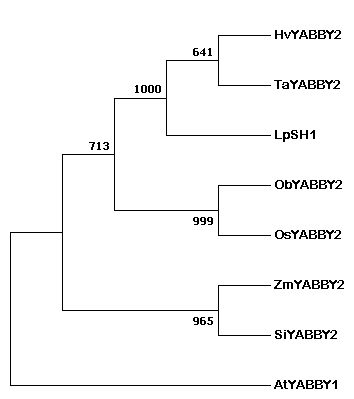

**Figure S7**. Rooted phylogenetic tree of the YABBY2 sub-family in monocots.

The tree is rooted using a homologous gene sequence in arabidopsis. The accession number are as follows: HvYABBY2 (*Hordeum vulgare*, BAJ89435.1); SiYABBY2 (*Setaria italic,* XP_004982272.1); ObYABBY2 (*Oryza brachyantha,* XP_006650352.1); TaYABBY2 (*Triticum aestivum,* ABW80974.1); OsYABBY2 (*Oryza sativa,* XP_015628574.1); ZmYABBY2 (*Zea mays,* XP_008666788.2); AtYABBY1 (*Arabidopsis thaliana,* AAD33715.1). Split was identified in 1000 bootstrap replicates.
